# Fracture Patterns in Type 1 and Type 2 Diabetes Mellitus: A Narrative Review of Recent Literature

**DOI:** 10.1007/s11914-021-00715-6

**Published:** 2021-12-21

**Authors:** V. Van Hulten, Nicklas Rasmussen, J.H.M. Driessen, A.M. Burden, A. Kvist, J.P. van den Bergh

**Affiliations:** 1 Department of Clinical Pharmacy and Toxicology, Maastricht University Medical Centre+ (MUMC+), Maastricht, The Netherlands; 2 NUTRIM School of Nutrition and Translational Research in Metabolism, Maastricht University, Maastricht, The Netherlands; 3 Cardiovascular Research Institute Maastricht, Maastricht University, Maastricht, The Netherlands; 4 Steno Diabetes Center North Denmark, Aalborg, Denmark; 5 Department of Chemistry and Applied Biosciences, Institute for Pharmaceutical Sciences, ETH Zurich, Zurich, Switzerland; 6 Department of Endocrinology and Metabolism, Molecular Endocrinology & Stem Cell Research Unit (KMEB), Odense University Hospital, Odense, Denmark; 7 Department of Internal Medicine, Division of Rheumatology, Maastricht University Medical Centre+ (MUMC+), Maastricht, The Netherlands; 8 Department of Internal Medicine, Subdivision of Endocrinology, VieCuri Medical Center, Venlo, The Netherlands

**Keywords:** Diabetes mellitus, Fracture risk, Glucose-lowering medication, Micro- and macrovascular complications

## Abstract

**Purpose of Review:**

In this narrative review, we have summarized the literature on fracture risk in T1DM and T2DM with a special focus on fracture site, time patterns, glucose-lowering drugs, and micro- and macrovascular complications.

**Recent Findings:**

T1DM and T2DM were associated with an overall increased fracture risk, with preferent locations at the hip, vertebrae, humerus, and ankle in T1DM and at the hip, vertebrae, and likely humerus, distal forearm, and foot in T2DM. Fracture risk was higher with longer diabetes duration and the presence of micro- and macrovascular complications. In T2DM, fracture risk was higher with use of insulin, sulfonylurea, and thiazolidinediones and lower with metformin use.

**Summary:**

The increased fracture risk in T1DM and T2DM concerns specific fracture sites, and is higher in subjects with longer diabetes duration, vascular complications, and in T2DM with the use of specific glucose-lowering medication.

**Supplementary Information:**

The online version contains supplementary material available at 10.1007/s11914-021-00715-6.

## Introduction

Diabetes mellitus is defined as a disease of inadequate control of blood glucose levels [1]. Both the prevalence of type 1 diabetes mellitus (T1DM) and type 2 diabetes mellitus (T2DM) are increasing worldwide [2, 3]. In 2015, the international diabetes foundation already estimated that most countries devote 5–20% of total healthcare expenditures to diabetes [4]. Moreover, diabetes is a major health threat and it has risen to the 12th cause of death in men, and even the sixth in women [5].

Both T1DM and T2DM are associated with microvascular and macrovascular complications [6, 7]. Additionally, both types have been reported to be associated with an increased fracture risk, while bone mineral density (BMD) was most often reported to be decreased in T1DM but not in T2DM [8]. Thus, while a decrease in BMD could underlie the increased fracture risk in T1DM, this does not seem to be the case for T2DM. In fact, for a long time, subjects with T2DM were not considered to be at risk for osteoporosis, unlike T1DM subjects [7•]. The development of bone fragility in T1DM and T2DM may be differential and can only partly be explained by BMD. Other factors such as bone microarchitectural deterioration that is not depicted by BMD measurements and fall risk may also contribute to fracture risk in T1DM and T2DM, although the importance of underlying factors may be different [9]. It is therefore likely that the incidence of fractures at various sites may be different in subjects with diabetes when compared to subjects without diabetes and may also be different between T1DM and T2DM due to differences in glucose-lowering medication use, differences in falling patterns, and differences in BMI. For example, the risk of hip fracture has generally been found to be higher in T1DM subjects compared to T2DM subjects [10], while obese women were found to be at a significantly higher risk of proximal humerus fracture than women of lower weight [11], which puts the T2DM population at particular risk.

Several studies reported on the risk of fracture in subjects with T1DM and T2DM. For T1DM, the risk of any, hip, and vertebral fractures was found to be increased in most [8, 12–18], but not all studies [19]. Information on other fracture sites in T1DM is scarce. In T2DM, overall fracture risk was reported to be increased [8, 12, 20–23], especially in women, and to a lesser extent or even no increased risk in men [17]. Most studies on fracture risk in T1DM and T2DM focused on hip, vertebral, or a combination of fracture sites. Given the potential difference in fracture risk among T1DM and T2DM at various sites, we have summarized the fracture pattern in T1DM and T2DM based on the risk of fracture at different sites and diabetes duration. Subsequently, we have summarized the risk associated with the use of glucose-lowering medication and with the presence of micro- and macrovascular complications.

For the purpose of this narrative review, we have used the umbrella term ‘distal forearm fractures’ to include fractures of the wrist and radius, and ‘humerus fractures’ to include all upper arm fractures. Furthermore, we focused our search for literature on fracture sites on studies published in or after 2015, in order to provide a summary of current knowledge in the field. Literature on the other covered topics was sparser, and therefore not as restricted. A further clarification of the methodological approach can be found in Supplementary material 1.

## Fracture Site Patterns

### T1DM

Table 1 summarizes the literature on the risk of fractures in T1DM, stratified by fracture site. We found an elevated risk of any fracture in T1DM [10, 18, 24, 25], as was the case for hip fractures [10, 18, 24–26]. The risk of vertebral fractures was only investigated by Shah et al. [18], who reported an increased risk (RR 2.88; 95% CI 1.71–4.82). Furthermore, the risk of distal forearm, humerus, and ankle fractures was studied by Wallander et al. [10], who reported an increased risk for humerus (HR 1.34; 95% CI 1.23–1.46) and ankle fractures (HR 1.49; 95% CI 1.29–1.71), but not for risk of distal forearm fractures. Lastly, a recent article described a probable increase in atypical femoral fractures in subjects with T1DM [27].

**Table 1:** Summary of previous literature on fracture sites in T1DM

Author	Number of subjects	Any (RR/HR (95% CI))	Hip (RR/HR (95% CI))	Vertebral (RR (95% CI))	Distal forearm RR/HR (95% CI))	Humerus (RR/HR (95% CI))	Ankle (RR/HR (95% CI))
Thong et al., 2018^1,3^	2,490,941	1.88 (1.5–2.32)*	4.40 (2.58–7.50)*				
Shah et al., 2015^1,3^	4,391,425	3.16 (1.51–6.63)*	3.78 (2.05–6.98)*	2.88 (1.71–4.82)*			
Fan et al., 2016^1,3^	4,515,811		5.76 (3.66–9.07)*				
Bai et al., 2020^1,3^	889,257	1.3 (1.2–1.4)*	5.3 (3.4–8.3)*				
Wallander et al., 2016^2^	349,146	1.33 (1.22–1.45)*	1.38 (1.21–1.58)*		0.93 (0.61–1.41)	1.34 (1.23–1.46)*	1.49 (1.29–1.71)*

*T1DM* type 1 diabetes, *RR* relative risk, *HR* hazard ratio, *95*% *CI* 95% confidence interval

*Denotes significant findings

^1^Study reports relative risk (RR)

^2^Study reports hazard ratio (HR)

^3^Meta-analysis

Additionally, it appears that subjects with T1DM have an increased risk of fractures at a younger age. In fact, a study by Weber et al. [28] found a modest increase of the occurrence of any fracture in subjects with T1DM aged 0–19 year. Furthermore, in another study, an increased risk for any fractures was found in subjects with T1DM between 18 and 50 years of age (RR 1.88; 95% CI 1.52–2.32) [24].

### T2DM

Overall, T2DM was associated with an increased risk of any fracture, with reported relative risks ranging from 1.02 [10] to 1.7 [25] (Table 2). Several meta-analyses have reported an increased risk of hip fractures [7•, 29, 26, 30, 25] in the T2DM population. However, the literature on vertebral fractures is less consistent. While Moayeri et al. [7•] reported an increased risk, Jia et al. [29] reported an equal risk. Furthermore, a meta-analysis including 15 studies showed a lower odds of prevalent (OR 0.84; 95% CI 0.74–0.95) but a higher odds of incident vertebral fractures (OR 1.35; 95% CI 1.27–1.44) [31].

**Table 2:** Summary of previous literature on fracture sites in T2DM

Author	Number of subjects	Any (RR/HR (95% CI))	Hip (RR/HR (95% CI))	Vertebral (RR (95% CI))	Distal forearm (RR (95% CI))	Humerus (RR (95% CI))	Ankle (RR (95% CI))	Foot (RR (95% CI))
Moayeri et al., 2017^1,3^	5,815,277	1.05 (1.04–1.06)*	1.20 (1.17–1.23)*	1.16 (1.05–1.28)*	0.98 (0.88–1.07)	1.09 (0.86–1.31)	1.13 (0.95–1.32)	1.37 (1.21–1.54)*
Jia et al., 2017^1,3^	938,742	1.23 (1.12–1.35)*	1.08 (1.02–1.15)*	1.21 (0.98–1.48)				
Fan et al., 2016^1,3^	4,687,867		1.34 (1.19–1.51)*					
Rasmussen et al., 2021^1^	814,018		1.02 (1.01–1.04)*		1.39 (1.18–1.61)*	1.24 (1.12–1.37)*		
Bai et al., 2020^1,3^	1,234,536	1.7 (1.1–2.7)*	1.6 (1.4–1.8)*					
Wallander et al., 2016^2,3^	422,762	1.02 (0.98–1.06)	1.03 (0.96–1.10)		0.70 (0.56–0.86)*	1.17 (1.01–1.35)*	1.01 (0.80–1.27)	

*T2DM* type 2 diabetes, *RR* relative risk, *HR* hazard ratio, *95*% *CI* 95% confidence interval

*Denotes significant findings

^1^Study reports relative risk (RR)

^2^Study reports hazard ratio (HR)

^3^Meta-analysis

Few studies are available on fracture risk at other sites, such as the distal forearm, humerus, ankle, and foot. Moayeri et al. [7•] has published a meta-analysis and found a similar risk in T2DM and controls of fractures at the distal forearm, humerus, and ankle, while the risk of fractures of the foot was increased in T2DM (RR 1.37; 95% CI 1.21–1.54). However, a later cohort-study by Rasmussen et al. [28] reported an increased risk of fracture in T2DM at the distal forearm (RR 1.39; 95% CI 1.18–1.61) and humerus (RR 1.24; 95% CI 1.12–1.37). Contrastingly, Wallander et al. [10] reported a decreased risk of distal forearm fractures (HR 0.70; 95% CI 0.56–0.86), an increased risk of humerus fractures (HR 1.17; 95% CI 1.01–1.35), and a similar risk for ankle fractures. However, the cohort studied by Wallander et al. [10] had a mean age of 80.8 ± 8.2 years old, while the mean age in the previously mentioned studies was generally much lower.

### T1DM and T2DM

Two meta-analyses focusing on diabetes in general, meaning both T1DM and T2DM, showed that fracture risk in diabetes mellitus (DM) is increased for any fracture [12, 25]. Additionally, the previously addressed increased risk of hip fracture was also reported in studies looking at T1DM and T2DM combined [12, 25, 26], although the risk of hip fractures was higher in T1DM compared to T2DM [12, 25, 26]. The risk of vertebral fractures in diabetic subjects was similar to controls in the two meta-analyses [12, 25]. The risk of distal forearm fractures has been reported to be unassociated with DM [12, 32], while risk of ankle fractures [12, 32] and humerus fractures [12] were reported to be increased in DM.

## Diabetes Duration

### T1DM

Longer diabetes duration has been associated with a higher risk of fracture in T1DM by some [33–35] but not all studies [36, 37]. Vestergaard et al. [33] observed a modest increase in risk of any fracture after > 5 years of diabetes duration, while Dhaliwal et al. [34] found that participants with a fracture more often had a longer diabetes duration. Additionally, Leanza et al. [35] reported an increased risk of ≥ 2 fractures for T1DM subjects with a disease duration of ≥ 26 years, compared to ≤ 14 years. However, both Joshi et al. [36] and Zhukouskaya et al. [37] did not find a significant effect of diabetes duration on risk of any fracture, or risk of vertebral fractures, respectively.

### T2DM

Longer duration of T2DM has been associated with an increased risk of fracture in most studies [7, 14, 17, 22, 38, 39]. Ahmed et al. [17] reported an increasing risk of hip fractures by increasing disease duration, similar to the results from the study by Schwartz et al. [39], who found that women who had been diagnosed with T2DM at least 14 years ago had a significantly a higher risk of hip fracture than diabetics with a shorter time since diagnosis. Furthermore, Moayeri et al. [7•] reported a higher risk of incident fractures for ≥ 10 years of diabetes duration, compared to that of ≤ 10 years of diabetes duration. Likewise, Ivers et al. [38] observed a similar result, where ≥ 10 years of diabetes duration was significantly associated with proximal humerus fractures. Additionally, both Janghorbani et al. [22] and Nicodemus et al. [14] found that fracture risk increased with longer duration of T2DM. However, Strotmeyer et al. [40] was not able to show a significant association between fracture risk and diabetes duration.

### T1DM and T2DM

Studies focusing on DM in general also show that longer diabetes duration increases fracture risk [41, 42]. Interestingly, Leslie et al. [41] found that long-term diabetes was associated with an increased risk of fracture, while newly diagnosed diabetes showed a reduction in fracture prevalence.

## Glucose-Lowering Medication

### Metformin

Hidayat et al. [43•] reported a reduced risk of fractures in metformin users compared to users of other oral glucose-lowering drugs (RR 0.86; 95% CI 0.75–0.99) in a meta-analysis based on 12 studies (Table 3), as did Tseng [44] in a cross-sectional study, who also found a significantly decreased risk of osteoporosis and vertebral fractures for metformin users (RR 0.59; 95% CI 0.55–0.64). However, a randomized controlled study performed by Nordklint et al. [45] showed that neither trabecular bone score nor bone mineral density differed significantly between metformin users and the placebo group, leaving the mechanism underlying the decreased fracture risk unelucidated. Furthermore, metformin is the first line treatment for T2DM, meaning that metformin use could be a reflection of a less severe or shorter duration of DM, and the decreased fracture risk might thus be partly attributable to the lower overall risk of fracture among metformin users [43•].

**Table 3:** Glucose-lowering drugs and fracture risk

Medication type	Study (type)	Number of subjects	Control group	Fracture risk (RR/HR (95% CI))
Metformin	Hidayat et al., 2019 (meta-analysis of observational studies)^1^	1,267,637	Not specified	0.86 (0.75–0.99)*
	Tseng et al., 2021 (cohort study)^1^	29,222	New-onset T2DM subjects	0.59 (0.55–0.64)*
Sulfonylureas	Zhang et al., 2020 (meta-analysis of RCTs and observational studies)^1^	255,644	T2DM subjects on placebo or active comparator	1.14 (1.08–1.19)*
	Hidayat et al., 2019 (meta-analysis analysis of observational studies)^1^	674,760	Not specified	1.30 (1.18–1.43)*
Insulin	Hidayat et al., 2019 (meta-analysis analysis of observational studies)^1^	4,594,081	Not specified	1.49 (1.29–1.73)*
	Losada-Grande et al., 2017 (cohort study)^2^	53,853	T2DM subjects	1.38 (1.06–1.80)*
	Corrao et al., 2020 (cohort study)^2^	54,998	T2DM subjects on oral glucose-lowering drugs	1.5 (1.3–1.6)*
	Zhang et al., 2019 (meta-analysis of case-control studies)^1^	138,690	T2DM subjects on oral glucose-lowering drugs	1.24 (1.07–1.44)*
SGLT2 inhibitors	Tang et al., 2016 (meta-analysis of RCTs)^3^	30,384	T2DM subjects on placebo	Canagliflozin: 1.15 (0.71–1.88) Dapagliflozin: 0.68 (0.37–1.25) Empagliflozin: 0.93 (0.74–1.18)
	Ruanpang et al., 2017 (meta-analysis of RCTs)^1^	8,286 treated with SGLT2 inhibitors	T2DM subjects on placebo	0.67 (0.42–1.07)
	Li et al., 2019 (meta-analysis of RCTs)^1^	20,895	T2DM subjects on placebo	1.02 (0.81–1.28)
	Cheng et al., 2019 (meta-analysis of RCTs)^3^	23,372	T2DM subjects on placebo	0.86 (0.70–1.06)
	Azharuddin et al., 2018 (meta-analysis of RCTs)^3^	32,343	T2DM subjects on placebo or active comparator	1.01 (0.83–1.23)
	Watts et al., 2016 (meta-analysis of case-control trials)^2^	10,194	T2DM subjects on placebo or active comparator	Canagliflozin: 1.32 (1.00–1.74)
DPP-4 inhibitors	Fu et al., 2016 (meta-analysis of RCTs)^1^	62,206	T2DM subjects on placebo or active comparator	0.95 (0.83–1.10)
	Mamza et al., 2016 (meta-analysis of case-control trials)^3^	36,402	T2DM subjects on placebo or active comparator	Placebo: 0.82 (0.57–1.16) active comparator: 1.59 (0.91–2.80)
	Yang et al., 2017 (meta-analysis of RCTs)^3^	8,218	T2DM subjects on placebo	Alogliptin: 0.51 (0.29–0.88)* (through network meta-analysis)
	Monami et al., 2011 (meta-analysis of RCTs)^3^	21,055	T2DM subjects on placebo or active comparator	0.60 (0.37–0.99)*
GLP-1 receptor agonists	Hidayat et al., 2019 (meta-analysis of observational studies)^1^	Not reported	Not specified	0.65 (0.24–1.74)
	Zhang et al., 2018 (meta-analysis of RCTs)^1^	49,602	T2DM subjects on placebo	Exenatide: 0.17 (0.03–0.67)*
	Su et al., 2015 (meta-analysis of RCTs)^3^	11,206	Non specified subjects on placebo or active comparator	Exenatide: 2.09 (1.03–4.21)* Liraglutide: 0.38 (0.17–0.87)*
Thiazolidinediones	Hidayat et al., 2019 (meta-analysis of observational studies)^1^	2,559,628	Not specified	1.24 (1.13–1.35)
	Schwartz et al., 2015 (cohort study)^2^	6,865	T2DM subjects on a lower TZD dose	1–2 years of TZD use: 2.32 (1.49–3.62)* > 2 years of TZD use: 2.01 (1.35–2.98)*
	Bazelier et al., 2013 (meta-analysis of cohort studies)^2^	1,637,084	T2DM subjects on other glucose-lowering drugs	Women: 1.44 (1.35–1.53)* Men: 1.05 (0.96–1.14)
	Loke et al., 2009 (meta-analysis of RCTs)	13,715	T2DM subjects not on thiazolidinedione therapy	Women: 2.23 (1.65–3.01)* Men: 1.0 (0.73–1.39) Overall: 1.45 (1.18–1.79)*
	Zhu et al., 2014 (meta-analysis of RCTs)	24,544	T2DM subjects not on thiazolidinedione therapy	Women: 1.94 (1.60–2.35)* Men: 1.02 (0.83–1.27)

*T2DM* type 2 diabetes, *RR* relative risk, *HR* hazard ratio, *95*% *CI* 95% confidence interval, , *DPP*-*4* dipeptidyl peptidase 4, *SGLT2* sodium–glucose cotransporter 2, *TZD* Thiazolidinediones

*Denotes significant findings

^1^Study reports relative risk (RR)

^2^Study reports hazard ratio (HR)

^3^Study reports odds ratio (OR)

### Sulfonylurea

Both Hidayat et al. [43•] and Zhang et al. [46] reported a higher relative risk for sulfonylurea use (RR 1.30; 95% CI 1.18–1.43) and RR 1.14; 95% CI 1.08–1.19, respectively). In subgroup analyses, the risk for sulfonylurea users compared to metformin users remained higher (OR 1.25; 95% CI 1.18–1.32), while the risk compared to insulin users was reported to be lower (OR 0.81; 95% CI 0.74–0.89).

### Insulin

Because the vast majority of T1DM subjects are insulin users, it is not feasible to study the association of insulin with fracture risk in T1DM, and therefore we focus our discussion on T2DM. A meta-analysis comprising 23 studies including subjects with T1DM and T2DM by Hidayat et al. [43•] showed a higher relative risk of fractures in insulin users compared to non-insulin users (RR 1.49; 95% CI 1.29–1.73) (Table 3).

A cohort study including subjects with T2DM using insulin, either in combination with oral glucose-lowering drugs or exclusively, by Losada-Grande et al. [47] showed an increased adjusted subhazard ratio for major fractures (HR 1.38; 95% CI 1.06–1.80). In line with these results, Corrao et al. [48] reported that subjects with T2DM who switched from oral glucose-lowering drugs to insulin had an increased risk of any fracture, hip fractures, and vertebral fractures. Both Hidayat et al. [43•] and Corrao et al. [48] suggested episodes of hypoglycemia and associated falls as a possible explanation for this observed immediate increased fracture risk. Finally, a meta-analysis of observational studies with 138,690 subjects with T2DM showed that insulin treatment was significantly associated with an increased risk of fracture (RR 1.24; 95% CI 1.07–1.44) as compared to oral glucose-lowering drugs [49•].

### Sodium–Glucose Cotransporter 2 Inhibitors

Five out of the six included meta-analyses [50–54] did not find a significant association of sodium–glucose cotransporter 2 (SGLT2) inhibitor use and fracture risk in T2DM (Table 3). Additionally, when looking at the different subclasses of SGLT2 inhibitors, specifically canagliflozin, dapagliflozin, empagliflozin, no significant increase or decrease of fracture risk was found in the aforementioned studies either. However, a meta-analysis by Watts et al. [55] did find a borderline significantly increased risk of fractures for canagliflozin treatment compared to placebo (HR 1.32; 95% CI 1.00–1.74), although this seemed to be driven by subjects who were older, with a prior history/risk of cardiovascular disease, and with lower baseline estimated glomerular filtration rate (eGFR) and higher baseline diuretic use.

### Dipeptidyl Peptidase 4 (DPP-4) Inhibitors

For DPP-4 inhibitor use in T2DM subjects, varying results were found. Two meta-analyses by Fu et al. [56] and Mamza et al. [57] reported no significant difference in fracture risk comparing DPP-4 inhibitor use versus either placebo or active comparators (Table 3). In the meta- analyses by Yang et al. [58] investigating subclasses of DPP-4 inhibitors separately, a decreased fracture risk for Alogliptin was found (OR 0.51; 95% CI 0.29–0.88), and the meta-analysis of Monami et al. [59], including all DPP-4 inhibitors, additionally showed a significantly decreased fracture risk (OR 0.60; 95% CI 0.37–0.99).

### GLP1 Receptor Agonists

The meta-analysis by Hidayat et al. [60•] reported that the use of GLP1 receptor agonists (GLP1 RAs) was not significantly associated with fracture risk (Table 3). However, they did find a significantly decreased risk for hip fractures specifically (RR 0.21; 95% CI 0.04–0.98). Furthermore, when looking at the different subtypes of GLP-1 RAs, Zhang et al. [61] found that fracture risk was lower in exenatide users (RR 0.17; 95% CI 0.03–0.67) relative to placebo. However, the meta-analysis performed by Su et al. [62] reported opposing results, and stated that exenatide was associated with a significantly increased risk of fractures compared to placebo or other active comparators (OR 2.09; 95% CI 1.03–4.21). Yet, this study was based on 2681 T2DM subjects and 2613 controls, as opposed to 49,602 participants in the meta-analysis by Zhang et al. [61, 62]. Additionally, Su et al. [62] found a significantly decreased risk of fracture for liraglutide compared to placebo or other active comparators (OR 0.38; 95% CI 0.17–0.87).

### Thiazolidinediones

The use of thiazolidinediones (TZDs) was associated with an increased fracture risk according to a meta-analysis by Hidayat et al. (RR 1.24, 95% CI 1.13–1.35) (Table 3) [43•]. Additionally, a cohort study by Schwartz et al. [21] showed that the fracture rate was higher in women with 1–2 years of TZD use or over 2 years of TZD use (HR 2.01; 95% CI 1.35–2.98) compared to non-users. Lastly, Bazelier et al. [63] performed a meta-analysis and found an increased risk of any fracture for women (HR 1.44; 95% CI 1.35–1.53) but not for men (HR 1.05; 95% CI 0.96–1.14). Additionally, their study showed an increased risk of fractures in women of the distal forearm, humerus, tibia/fibula, ankle, and foot, but not for hip/femur or vertebral fractures. This was in line with the findings from the meta-analyses of RCTs by Loke et al. [64] and Zhu et al. [65], who both found an increased fracture risk with TZD use for women (OR 2.23; 95% CI 1.65–3.01 and OR 1.94; 95% CI 1.60–2.35, respectively), but not for men.

## Microvascular and Macrovascular Complications of Diabetes

Diabetic peripheral neuropathy (DPN) is characterized by axonal degeneration and segmental demyelination, affecting the nerve fibers and motor nerves [66, 67]. It is the most common complication of diabetes, although it has been reported to functionally affect T1DM subjects more severely compared to T2DM subjects [68]. The influence of DPN on fracture risk also seemed to be slightly more pronounced in T1DM subjects compared to T2DM subjects, as was shown in a meta-analysis by Liu et al. [69•] which reported a higher OR for fractures in subjects with T1DM and T2DM with DPN compared to without DPN (T1DM: OR 2.43 (95% CI 1.61–3.68), T2DM: OR 2.15 (95% CI 1.56–2.97)). The ORs for T1DM and T2DM were not significantly different. Additionally, a cohort study showed an increased risk of incident fractures for T1DM subjects with DPN compared to those without, in both males (OR 1.42; 95% CI 1.01–1.99) and females (OR 1.98; 95% CI 1.43–2.75) [28]. Furthermore, two studies in subjects with T2DM in the Asian population also found that the presence of DPN was associated with an increased odds of fracture, with an OR 9.51 and even 37.3, respectively [70, 71]. However, the 95% confidence intervals in both these studies were very wide. Contrastingly, a study by Kahn et al. [72] in a Caucasian population was unable to find a significant association between DPN and fracture prevalence, as determined by data-extraction from the Danish National Patient Register. Additionally, a cohort study including both T1DM and T2DM reported a significant association between DPN and major osteoporotic fractures (HR 2.52; 95% CI 1.41–4.50) for Caucasians, but interestingly this significant association was not seen in the African American or Hispanic population [73•]. Lastly, in a retrospective study performed by Lee et al. [74•], fracture risk for any fracture (RR 1.17; 95% CI 1.16–1.18), but also specifically for hip fractures (RR 1.16; 95% CI 1.15–1.18), was reported to be increased, and DPN was stated to be the most important mediator of fracture risk observed in their cohort. Therefore, we can conclude that fracture risk is higher in subjects with DPN, both in T1DM and T2DM.

Sparse knowledge exists on the association between diabetic eye disease (DED) and the risk of fractures. DED is a common microvascular diabetic complication and affects approximately 30% of subjects with diabetes. The prevalence is twice as high in T1DM compared to T2DM [75, 76]. However, studies performed on the association between DED and fracture risk mainly included subjects with T2DM, except for the cohort study by Weber et al. [28], who included T1DM subjects and reported an OR of 1.29 (95% CI 1.08–1.55) for subjects with retinopathy compared to subjects without. The observational study by Yokomoto-Umakoshi et al. [70] included T2DM subjects and reported no significant association between retinopathy and the risk of fractures. In contrast, an observational study in postmenopausal women by Viegas et al. [77] reported that the frequency of fractures was significantly associated with the presence of diabetic retinopathy (*P* value = 0.030). The retrospective study by Jain et al. [73•] evaluated the association of DED with fracture risk among different ethnicities and found that only in the Hispanic DM population, DED was associated with a borderline increased fracture risk (HR 2.12; 95% CI 1.00–4.46), while this was not the case for the Caucasian and African American population. Lastly, Ivers et al. [38] analyzed the association between DED and specific fracture sites, and found that although DED was significantly associated with any fracture (RR 5.4; 95% CI 2.7–10.8), a significant site-specific association could only be found for proximal humerus fractures (RR 10.3; 85% CI 2.2–48.0), but not for hip, distal forearm, or ankle fractures.

Thus, generally speaking, DED significantly increases the risk of any fracture in both the T1DM and T2DM population, although this cannot be extrapolated to all subpopulations or fracture sites. Further studies are needed to provide a definitive answer on this matter.

Diabetic kidney disease (DKD) or nephropathy is a significant cause of chronic kidney disease and end-stage renal failure globally [78]. Nephropathy generally develops after many years in T1DM, while it may already be present at the time of diagnosis of T2DM [78, 79]. A recent meta-analysis found that a lower eGFR was associated with a significantly higher risk of all fractures (HR 2.63; 95% CI 1.74–3.98) and hip fractures (HR 1.36; 95% CI 0.99–1.86) [80•]. Interestingly, a graded risk was observed, with higher risk among the more severe stages of DKD. The retrospective study by Lee et al. [74•] reported similar results, namely a significantly increased risk for both any fracture (RR 1.2; 95% CI 1.20–1.22) and hip fractures (RR 1.20; 95% CI 1.18–1.21). However, although the increased risk of fracture with DKD was observed by Jain et al. [73•] in the African American population (HR 2.05; 95% CI 1.11–3.79), this was not the case for the Caucasian or Hispanic population included in this study. Furthermore, two studies by Yokomoto-Umakoshi et al. [70] and Viegas et al. [77] were both unable to show a significant association between fracture risk and nephropathy. Hence, the association between fracture risk and DKD remains to be elucidated.

In general, diabetes is a well-known risk factor for developing macrovascular complications [81]. Jain et al. [73•] did not observe an increased risk of fractures associated with either ischemic heart disease, atrial fibrillation, or congestive heart failure in Caucasians, African Americans, or Hispanics. Additionally, Yokomoto-Umakoshi et al. [70] obtained similar non-significant results in a Japanese cohort of T2DM subjects. However, Lee et al. [74•] analyzed a cohort of male T2DM subjects, and did find an increased risk of fractures in subjects with T2DM with cardiovascular disease in general (OR 1.18; 410 95% CI 1.17–1.20) and congestive heart failure (OR 1.18; 95% CI 1.16–1.19). Therefore, we can conclude that there is no clear indication for an association between macrovascular disease and fracture risk in T2DM.

## Conclusions

T1DM and T2DM were associated with an overall increased fracture risk, with preferent locations at the hip, vertebrae, humerus, and ankle in T1DM and at the hip, vertebrae, and likely humerus, distal forearm, and foot in T2DM. Fracture risk was higher with longer diabetes duration and the presence of micro- and macrovascular complications. In T2DM, fracture risk was higher with use of insulin, sulfonylurea, and thiazolidinediones and lower with metformin use.

A factor that may be important with regard to the explanation of the differing preferent fracture sites in T1DM and T2DM could be the differences in body composition. Subjects with T2DM are often overweight or obese, and it is suggested that obesity reduces the risk of distal forearm fractures while it increases the risk of ankle fractures [82]. Furthermore, obese women were found to be at a significantly higher risk of proximal humerus fracture than women of lower weight [11]. This might be due to a different falling pattern between obese subjects and their non-obese counterparts. In fact, it has been suggested that obese subjects generally have an altered recovery response when tripping, which could increase impact force with the ground [83]. In contrast, additional padding at the hip as a consequence of overweight or obesity is likely to have a protective effect from hip-fractures [84].

Furthermore, the overall fall risk was found to be greater for T1DM subjects compared to T2DM subjects [85]. A possible reason for the increased fall risk of T1DM subjects compared to T2DM subjects could be the fact that T1DM subjects are more likely to suffer from microvascular complications, such as DPN and DED [75, 76].

Furthermore, while T1DM often goes paired with a decreased BMD, T2DM subjects are reported to have a normal or higher BMD [8]. However, it has been suggested that increasing BMI and percentage body fat can lead to precision errors in BMD measurement by DXA. Consequently, BMD measurement errors may be more likely in T2DM patients due to their generally higher BMI [86, 87]. Additionally, recent evidence obtained through high-resolution peripheral quantitative computed tomography scans suggests a decrease in volumetric BMD, as opposed to areal BMD, and an increase in cortical porosity in DM subjects [88–90]. A recently published overview by Van den Bergh et al. [91] summarized that in some, but not all studies in postmenopausal women, cortical porosity is greater in T2DM compared to controls, while additionally cortical BMD and cortical thickness have been reported to be decreased. This results in a decreased bone quality, and perhaps lead to a decreased bone turnover. This decreased peripheral bone quality might attribute to peripheral fractures in T2DM subjects, as proposed previously [12, 30, 32].

With regard to the use of medication, based on recent literature, insulin, sulfonylurea, and thiazolidinediones use was associated with increased fracture risk in T2DM, while metformin use was associated with a decreased fracture risk. SGLT2 inhibitor and DPP-4 inhibitor use was not significantly associated with fracture risk. The first line treatment for T2DM, which is metformin, has been associated with a decreased fracture risk [43•, 44]. However, sulfonylurea, the second most prescribed glucose-lowering drug in T2DM was reported to increase fracture risk. Furthermore, it is clear that insulin use was associated with an increased fracture risk [43•, 47, 48, 49•]. However, insulin is generally prescribed to T1DM, and to the more severe cases of T2DM, who often have (diabetes related) micro- and macrovascular complications. Therefore, we cannot differentiate whether the increased fracture risk associated with insulin use is due to the metabolic effects of insulin, or that it is a proxy of more severe T2DM.

Lastly, micro- and macrovascular complications of diabetes could contribute to the observed increase in fracture risk. Neuropathy contributes to the increased risk of fractures directly through effects on bone turnover and indirectly through effects on balance, muscle strength, and gait that increase the risk of falls. Furthermore, cardiovascular events as a consequence of macrovascular deterioration increase fall risk, and thus indirectly increase fracture risk.

However, the results should be interpreted with some caution. We have summarized the current knowledge, some of which is based on the results of observational studies. Observational studies carry some limitations, due to the fact that they are generally more prone to bias and confounding, and cannot be used to demonstrate causality [92]. On the contrary, clinical trials are more difficult to generalize, as the studied population might differ from the intended treatment population [93]. Furthermore, many of the included studies did not report biochemical measures, such as HbA1c, forcing us to base our conclusions solely on the presented data. Lastly, a number of the meta-analyses that were described in this review included studies with a follow-up duration of as little as 12 weeks.

To conclude, the increased fracture risk in T1DM and T2DM concerns specific fracture sites, and is higher in subjects with longer diabetes duration, vascular complications, and in T2DM with the use of specific glucose-lowering medication.

## Supplementary information

ESM 1 (DOCX 14.2 kb)
