# Supplementary material for: Fracture Patterns in Type 1 and Type 2 Diabetes Mellitus: A Narrative Review of Recent Literature
Source: Curr Osteoporos Rep. 2021 Dec 21;19(6):644–55. doi: 10.1007/s11914-021-00715-6 (PMC8716348; doi:10.1007/s11914-021-00715-6)
Supplement: Supplementary file 1 — (DOCX 14.2 kb) [file 11914_2021_715_MOESM1_ESM.docx]

**Appendix**

**Supplementary material 1: Clarification of the methods.**

To identify all relevant literature, the following search string was entered into the PubMed database on the 29^th^ of April: (“Diabetes Mellitus, Type 1”[Mesh] OR “Diabetes Mellitus, Type 2”[Mesh] OR diabet*[tw] OR tdm2[tw] OR dm2[tw] OR dm1[tw] OR tdm1[tw] OR t1dm[tw] OR t2dm[tw] OR 'type 1 diabetes*'[tw] OR 'Type 2 diabetes*'[tw] OR “t1d”[tw] OR “t2d”[tw]) AND ("Fractures, Bone"[Mesh][tiab]) AND (pattern[tw] OR site[tw] OR location[tw]) NOT (review[Publication Type]).

Additionally, the Embase database was searched on the 29^th^ of April using the Emtree terms ‘(diabetes:ti,ab,kw OR t1d:ti,ab,kw OR t2d:ti,ab,kw OR t1dm:ti,ab,kw OR t2dm:ti,ab,kw OR diabetic:ti,ab,kw OR 'diabetes melitus':ti,ab,kw OR 'type 1 diabetes':ti,ab,kw OR 'type 2 diabetes':ti,ab,kw) AND (fractures:ti,ab,kw OR bone:ti,ab,kw) AND (site:ti,ab,kw OR location:ti,ab,kw OR pattern:ti,ab,kw).

Articles were included if they described fractures at any site, or if the primary or secondary end-point of the study described fracture patterns or sites. Articles were excluded when abstracts were not published, the article described duplicated data from a previous publication, or concerned in vivo/in vitro animal studies. Furthermore, the full manuscript had to be available in either English, Danish or Dutch.
